# Support vector machine versus logistic regression modeling for prediction of hospital mortality in critically ill patients with haematological malignancies

**DOI:** 10.1186/1472-6947-8-56

**Published:** 2008-12-05

**Authors:** T Verplancke, S Van Looy, D Benoit, S Vansteelandt, P Depuydt, F De Turck, J Decruyenaere

**Affiliations:** 1 Department of Intensive Care Medicine, Ghent University Hospital, Faculty of Medicine, Ghent University, Ghent, Belgium; 2 Department of Information Technology, Faculty of Engineering, Ghent University, Ghent, Belgium; 3 Department of Applied Mathematics and Computer Science, Faculty of Sciences, Ghent University, Ghent, Belgium

## Abstract

**Background:**

Several models for mortality prediction have been constructed for critically ill patients with haematological malignancies in recent years. These models have proven to be equally or more accurate in predicting hospital mortality in patients with haematological malignancies than ICU severity of illness scores such as the APACHE II or SAPS II [1]. The objective of this study is to compare the accuracy of predicting hospital mortality in patients with haematological malignancies admitted to the ICU between models based on multiple logistic regression (MLR) and support vector machine (SVM) based models.

**Methods:**

352 patients with haematological malignancies admitted to the ICU between 1997 and 2006 for a life-threatening complication were included. 252 patient records were used for training of the models and 100 were used for validation. In a first model 12 input variables were included for comparison between MLR and SVM. In a second more complex model 17 input variables were used. MLR and SVM analysis were performed independently from each other. Discrimination was evaluated using the area under the receiver operating characteristic (ROC) curves (± SE).

**Results:**

The area under ROC curve for the MLR and SVM in the validation data set were 0.768 (± 0.04) vs. 0.802 (± 0.04) in the first model (p = 0.19) and 0.781 (± 0.05) vs. 0.808 (± 0.04) in the second more complex model (p = 0.44). SVM needed only 4 variables to make its prediction in both models, whereas MLR needed 7 and 8 variables in the first and second model respectively.

**Conclusion:**

The discriminative power of both the MLR and SVM models was good. No statistically significant differences were found in discriminative power between MLR and SVM for prediction of hospital mortality in critically ill patients with haematological malignancies.

## Background

**Figure 1:**
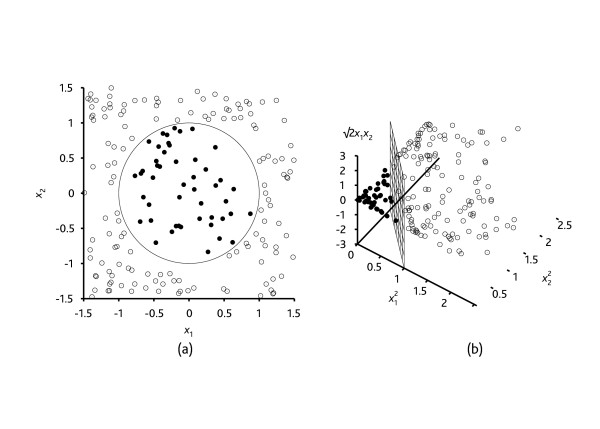
**Classification by a support vector machine algorithm is performed by transforming the input variables data set by means of a mathematical function into a higher dimensional input space in which separation is much easier.** The basis of this new heuristic is that classification of a seemingly chaotic input space is possible by increasing the dimensionality of that input space and thereby finding a separating boundary i.e. hyperplane. e.g.: (a) A two-dimensional training with positive examples as *black circles *and negative examples as *white circles*. The true decision boundary, (x1)^2 ^+(x2)^2 ^≤ 1, is also shown. (b) The same data after mapping into a three-dimensional input space ((x_1_)^2^, (x_2_)^2^, √2(x_1_)(x_2_)). The circular decision boundary in (a) becomes a linear decision boundary in three dimensions (b). (copyright permission from Prentice Hall).

Support vector machine (SVM) algorithms have not yet been studied for prediction of hospital mortality in the Intensive Care Unit (ICU). The SVM algorithm as a relatively new classification or prediction method, has been developed by Vapnik et al. in the 1990s [2-4] as a result of the collaboration between the statistical and the machine learning research community. The heuristic behind the SVM algorithm is quite different from that of the commonly used logistic regression (LR) modeling for prediction. This latter approach is the current standard for prognostic modeling in the ICU and is best known by clinicians. The LR algorithm uses a weighted least squares algorithm, i.e. the prediction is based on construction of a regression line as the best fit through the data points by minimizing a weighted sum of the squared distances to the fitted regression line. SVM, in contrast, tries to model the input variables by finding the separating boundary – called hyperplane – to reach classification of the input variables: if no separation is possible within a high number of input variables, the SVM algorithm still finds a separation boundary for classification by mathematically transforming the input variables by increasing the dimensionality of the input variable space. Figure 1 is very usefull for a thorough comprehension of the basics of this algorithm. In this figure, one can remark that the input variables cannot be separated in two dimensions, but very easily be separated in three dimensions. In the same way, the SVM algorithm can extrapolate this procedure mathematically to higher dimensions. The general term for a separating straight line in a high-dimensional space is a hyperplane. In clinical research, only a handful of articles have been published as a proof of concept of SVM [5,6], and none have been published till now for prediction of hospital mortality. This contrasts with the higher number of SVM publications in fundamental research such as bioinformatics [7] and genetics [8]. The main advantage of the SVM algorithm as a data mining method – which is a general term for the science of extracting useful information from large data sets or databases [9] -, is that it can more easily overcome the 'high dimensionality problem', i.e. the problem that arises when there is a high number of input variables relative to the number of available observations. Moreover, SVM has consistently shown to be a good classifier [10]. A nice introduction for the clinician to the basis of the SVM algorithm is the article by Noble et al. in Nature [11].

### Rationale

This study investigates the use of a support vector machine based classification model for determining the prognosis of ICU patients with haematological malignancies by comparing it with a logistic regression based classification model. The main goal of this article is to be a proof of concept for the use of SVM technology in the ICU, rather than to develop a predictive or prognostic model. Discrimination will be studied for both the MLR and the SVM classification models.

## Methods

### Data collection

The study was approved by the Ethics Committee of the Ghent University Hospital prior to the start of the data collection. Informed consent was waived because of the noninterventional study design. The data for this study were prospectively collected and consisted of an observational cohort of 352 patients with haematological malignancies admitted consecutively to the ICU between 1997 and 2006 for life-threatening complications. Patients admitted for monitoring, patients who received a do not resuscitate order before admission or patients admitted after elective or unscheduled surgery were excluded [12]. Of the original database of 372 patients only 20 patients were excluded because of 1 or more missing values, yielding n = 352 patients for definite analysis. Only the variables in the database which had shown to have an impact on hospital mortality in previous research were retained as well as those which were of potential clinical importance as indicated by a panel of experts [1,12]. Of the total of 352 patient records, 252 were used for training of the MLR and the SVM models, whereas 100 were used for validation. The 100 patients for the validation data set were selected so that the hospital mortality in the validation data set and the training data set was the same. Besides this criterion, the selection of patients for the training and validation data set was random. In a first model, 12 variables were included at the start of the modeling process for comparison between MLR and SVM (Table 1). In a second more complex model 17 variables were used (Table 2). The Acute Physiology and Chronic Health Evaluation II (APACHE II) score was incorporated in the first model but not in the second model. Clinically relevant physiological variables were included in the second model as a substitute for the APACHE II score. In this way, the authors investigated if a more simple model 1 with an APACHE score had better discriminative power than a more complex model 2 where the APACHE score was omitted but physiological patient variables were added. Regarding the physiological variables, the worst value of the first 24 h of ICU admission was retained in the data sets. Hospital mortality was determined as the main outcome variable. The performance in predicting hospital mortality with the APACHE II score [13], the Simplified Acute Physiology score II (SAPS II) and with the cancer specific severity of illness score (CSSI-score) (developed by Groeger and coworkers [14]) will be mentioned for comparison with the performance of the developed models. MLR and SVM analysis were performed independently from each other by different authors of this study. Statistical differences between the input variables of the training and the validation data set were examined with a Chi-square/Fisher's Exact test for categorical variables and a Mann-Whitney U test for continuous variables.

**Table 1:** Initial 12 input variables for model 1 before start of MLR and SVM modeling process and their descriptive statistics for the training and validation data sets.

**Input variable**	**Training**	**Validation**
gender, % male	58	65
age, yrs	55 (± 18)	58 (± 15)
% high-grade malignancy	61	54
% active disease of relapse	34	39
% allogeneic bone marrow transplant./stem cell transplant.	13	10
weeks since BMT, median (IQR)*	15 (54)	8 (102)
% chemotherapy<3 we since ICU admission	41	52
days of hospitalisation before ICU admission, median (IQR)	4(16)	6(16)
% bacterial infection	44	43
APACHE II score	24.5 (± 7.4)	25 (± 7.4)
% ventilated on day 1	49	46
% vasopressor need on day 1	41	49

training n = 252, validation n = 100; mean (± SD), except when indicated otherwise, *within subgroup with bone marrow transplantation.

**Table 2:** Initial 17 input variables for model 2 before start of MLR and SVM modeling process and their descriptive statistics for the training and validation data sets.

**Input variable**	**Training**	**Validation**
age, yrs	55 (± 18)	58 (± 15)
% high-grade malignancy	61	54
% active disease of relapse	34	39
% allogeneic bone marrow transplant./stem cell transplant.	13	10
days of hospitalisation before ICU admission, median (IQR)	4 (16)	6 (16)
% bacterial infection	44	43
pulse (/min)	123 (± 28)	118 (± 33)
mean blood pressure (MAP), mmHg	73 (± 27)	69 (± 22)
respiration frequency (/min)	32 (± 10)	33 (± 13)
Pa02/Fi02 (p/f)	198 (± 130)	194 (± 126)
platelets (1000/mm^3^)	125 (± 700)	90 (± 114)
urea<24 h (g/l)	0.86 (± 59)	0.82 (± 55)
creatinine<24 h (mg/dl)	1.6 (± 1.08)	1.7 (± 1.7)
albumin<24 h (g/dl)	2.6 (± 1.97)	2.4 (± 0.70)
prothrombin time (%)<24 h	56 (± 20.7)	57 (± 19.4)
% ventilated on day 1	49	46
% vasopressor need on day 1	41	49

training n = 252, validation n = 100; mean (± SD), except when indicated otherwise.

### Development of the logistic regression models for prediction of hospital mortality

Two MLR models were developed consecutively. The MLR model building processes were carried out in SPSS 16.0 (SPSS Inc., Chicago, Il, USA). In model one, 8 categorical variables and 4 continuous variables were included at the beginning of the analysis (Table 1). In model two, 6 categorical and 11 continuous variables were included at the beginning of the analysis (Table 2). To assess the relationship between a continuous variable and the outcome and to subsequently analyze whether transformation or categorization of continuous variables was needed, a scatterplot smoother and a method of quartiles was used for each variable. Forward and backward stepwise selection procedures were used. A p-value of 0.05 or less was considered to be significant for inclusion into the multivariable model. From the initial 12 (Table 1) and 17 variables (Table 2) in the univariate analysis in model 1 and 2 respectively, only 7 variables for model 1 and 8 variables for model 2 were significantly associated with the outcome in the multivariable analysis (Table 3 and 4, MLR model 1 and 2). The continuous variable 'platelets' in model 2 was dichotomized after scatterplot analysis (cut-off value for platelets at 50.000/mm3). In the same way, the variable 'urea' in model 2 was divided into 3 groups after scatterplot analysis (urea<0.5 g/l = reference category, 0.5 g ≤ urea<1 g/l and urea ≥ 1 g/l, respectively). The variables 'arterial oxygen pressure/fractional inspiratory oxygen (PaO2/FiO2) ratio' and the 'prothrombin time (%)' in model 2 were included as continuous variables since they had a linear relationship with hospital mortality. A correlation and a multicollinearity analysis were performed prior to the goodness of fit analysis. Clinically relevant interaction terms were added to the main effect model (e.g. gender*age), but none had statistically significant regression coefficients and hence none were retained in the final regression model. Table 3 and 4 show the final regression model on the training data set for model 1 and 2 respectively. The original parameter estimates from the training data set were then applied to the validation data set.

**Table 3:** MLR model 1: variables retained for final MLR analysis after variable selection process, coefficients, standard errors of the coefficients, odds ratios, 95% confidence intervals (CI) for the odds ratios for the model variables (x), and p-value.

**Variable**	**Coefficient**	**SE**	**Odds Ratio**	**95% CI**	**p-value**
gender*	-.636	.305	.530	.292–.962	0.037
high-grade malignancy	.689	.304	1.992	1.099–3.613	0.023
active disease	.797	.321	2.218	1.181–4.165	0.013
bone marrow transplant.	.914	.443	2.495	1.048–5.941	0.039
bacterial infection	-.739	.316	.478	.257–.887	0.019
APACHE II (per point)	.084	.024	1.088	1.037–1.140	0.001
ventilation < 24 h	1.221	.323	3.391	1.800–6.388	<0.001
Constant	-2.006	.707	.135		0.005

*female gender

**Table 4:** MLR model 2: variables retained for final MLR analysis after variable selection, coefficients, standard errors of the coefficients, odds ratios, 95% confidence intervals for the odds ratios for the model variables (x), and p-value

**Variable**	**Coefficient**	**SE**	**Odds Ratio**	**95% CI**	**p-value**
High-grade malignancy	.670	.324	1.954	1.034–3.690	0.039
active disease	.850	.328	2.340	1.229–4.456	0.010
bacterial infection	-781	.324	.458	.243–.863	0.016
thrombocytopenia (<50.000/mm^3^)	.867	.314	2.379	1.287–4.399	0.006
ventilation < 24 h	1.414	.327	4.111	2.167–7.798	<0.001
prothrombin time (%)	-0.016	.008	.984	.970–1.000	0.045
PaO2/FiO2 (p/f)	-.003	.001	.997	.995–1.000	0.025
urea < 0.5 g/l (reference)					0.011
urea 0.5–1 g/l	0.583	.415	1.791	.876–.3.663	0.033
urea > 1 g/l	1.249	.387	3.486	1.545–7.866	0.085
Constant	-0.457	.716	0.633		0.021

### Development of the support vector machine based models for prediction of hospital mortality

Two SVM models were developed consecutively. The SVM based model building processes were carried out with a modified Java version of the libSVM 2.82 software package available at . For both models the model construction process consisted consecutively of: (i) selection of the input variables (out of the 12 and 17 variables at the beginning of the modeling), (ii) selection of the training parameters (C and γ), (iii) construction of the model, (iv) performance evaluation and finally (v) validation of the model. The method of input variable selection was based on the approach in [15] which was itself based on the recursive feature elimination method as proposed in [16]. The common part in these approaches is that the input variables are ranked by iteratively eliminating the least important input variable in each step. In the approach used in this study, a second ranking is constructed by iteratively adding the most important input variable to the model. The libSVM training algorithm is a stochastic process, meaning that two consecutive runs do not necessary result in identical results. Therefore the rankings were repeated 160 times, after which the median ranking of each input variable was calculated. The last step in the input variable selection process, is to determine the exact number of input variables. In order to fix this number, the performance of the prediction model is estimated for an increasing number of input variables. This results in an initially increasing performance estimation, which after reaching a peak will decrease again. The number of input variables at peak estimated performance determines how much input variables are used in the final model. After the input variables for model construction are known, the model training parameters can be tuned. The SVM with a Gaussian kernel function has two such training parameters: C which controls overfitting of the model, and gamma (γ) which controls the degree of nonlinearity of the model. Gamma is inversely related to sigma which is a degree for spread around a mean in statistics: the higher the value of gamma, the lower the value of sigma, thus the less spread or the more nonlinear the behavior of the kernel. The values of these training parameters C and gamma are determined by grid search and cross validation: the model with the highest estimated performance determines the selected training parameters. Then, the performance of the constructed model is estimated by using 5-fold cross validation on the training data. Finally, the constructed model is validated by predicting the validation data and comparing these predictions with the real observations by means of ROC curves.

### Comparison between models

Comparison of MLR and SVM discrimination for both models was performed using SPSS 16.0 (SPSS Inc., Chicago, Il, USA) and SAS version 9.1.3 (macro %roc) (SAS Institute, Cary, NC, USA). Results are reported as percentages, means, minimums and maximums, ranges, and SDs (as appropriate). Accuracy (ACC), Sensitivity (SN), specificity (SP), positive and negative predictive values (PPV/NPV) for both models were calculated with 'R' free software version 2.6.1 (R Foundation for Statistical Computing) (Table 5). The cut-off value for calculation of these values was set by default at 0.5 (R-software 2.6.1 ROCR package). In the MLR analysis, the odds ratios were computed by taking e^x^, with x the value of the variable's coefficient in each of the two models (cf. Table 3 and 4). To test the ability of each model to distinguish patients who die from patients who live (discrimination), the area under the receiver operating characteristic curve (AUC) was calculated. Values above 0.80 indicate good discrimination [17]. To test the degree of correspondence between observed and predicted mortality over the entire range of risk (calibration), the Hosmer-Lemeshow (HL) goodness of fit statistic was calculated for the MLR models. To evaluate the statistical difference between the discrimination predicted by the MLR and SVM, a nonparametric test was used [18] with SAS version 9.1.3 (macro %roc).

**Table 5:** Accuracy (ACC), sensitivity (SN), specificity (SP), positive predictive value (PPV), negative predictive value (NPV) for model 1 and 2 for prediction of hospital mortality (95%CI)

	**MLR model1**	**SVM model1**	**MLR model2**	**SVM model2**
ACC	0.730 (0.632–0.814)	0.680 (0.579–0.770)	0.740 (0.643–0.823)	0.680 (0.579–0.770)
SN	0.740 (0.603–0.850)	0.630 (0.487–0.760)	0.722 (0.583–0.835)	0.630 (0.487–0.757)
SP	0.717 (0.565–0.840)	0.740 (0.589–0.857)	0.761 (0.612–0.874)	0.739 (0.589–0.857)
PPV	0.755 (0.617–0.862)	0.740 (0.589–0.857)	0.780 (0.640–0.885)	0.739 (0.589–0.857)
NPV	0.702 (0.551–0.827)	0.630 (0.487–0.757)	0.700 (0.554–0.821)	0.630 (0.487–0.757)

## Results

Hospital mortality in the training data set was 54.4% and 54.0% in the validation set.

**Figure 2:**
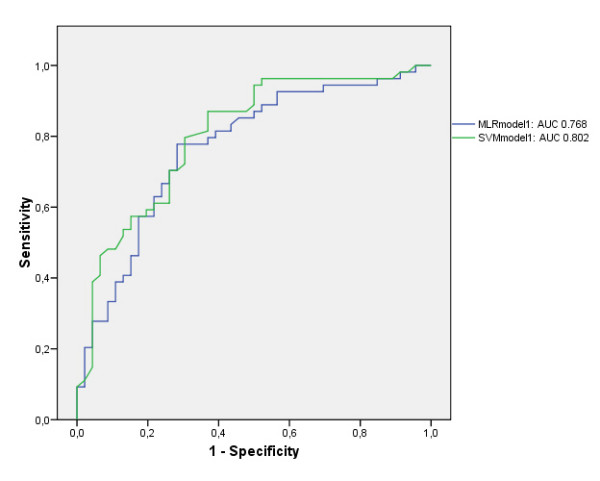
Area under the ROC curve (AUC) for comparison of the MLR and SVM in model 1.

**Figure 3:**
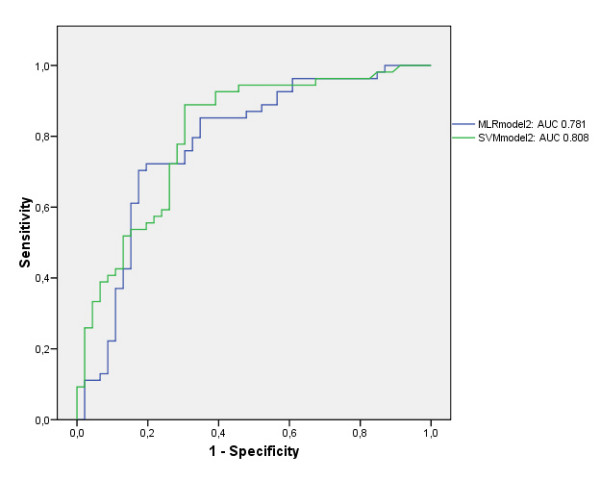
Area under the ROC curve (AUC) for comparison of the MLR and SVM in model 2.

Table 1 and 2 show a descriptive analysis of the initial input variables of the training and validation data set of model 1 (Table 1) and model 2 (Table 2). No statistically significant differences were found between the training and validation data sets. The AUC's for the training data set of model 1 were 0.791 (± 0.03) and 0.743 (± 0.04) for the MLR and SVM algorithm respectively, and 0.768 (± 0.04) and 0.802 (± 0.04) (p = 0.19) (Fig. 2) for the validation data set. The AUC's for the training data set of model 2 were 0.810 (± 0.03) and 0.723 (± 0.04) for the MLR and SVM algorithm respectively, and 0.781 (± 0.05) and 0.808 (± 0.04) for the validation data set (p = 0.44) (Fig. 3). The HL goodness of fit measures for the MLR training models had p-values of 0,932 (Chi-square 3.044; df 8) and 0.591 (Chi-square 6.504; df 8) in model 1 and 2 respectively. The HL goodness of fit measures for the SVM training models had p-values of <.001 (Chi-square 33.885; df 8) and <.001 (Chi-square 61.982; df 8) in model 1 and 2 respectively. In the MLR model 1 and 2, 7 and 8 variables were retained (Table 3 and 4). In contrast, in the SVM model 1 and 2, only 4 variables were retained: in model 1 these were 'ventilation < 24 h:Y/N', 'bone marrow transplantation:Y/N', 'bacterial infection:Y/N' and 'APACHE II-score', in model 2 these were 'ventilation < 24 h:Y/N', 'bone marrow transplantation:Y/N', 'bacterial infection:Y/N' and 'pulse'. The AUC's in the validation data set for the APACHE II score, the SAPS II score and the CSSI-score were 0,620 (± 0,06), 0.631 (± 0.06) and 0.727 (± 0.05) respectively. The accuracy, the sensitivity and specificity as well as the PPV and NPV of model 1 and 2 (corresponding to the optimal cut-off) for both the MLR and SVM models are mentioned in Table 5.

## Discussion

The equal distribution of survivors and nonsurvivors in the training and validation data sets makes logistic regression modeling ideal for comparison with other algorithms such as SVM [19]. Both the MLR an SVM models perform well in this study and the small differences between the MLR and SVM results in these data sets were statistically not significant. This is the first study to explore the future clinical use of SVM algorithms for mortality prediction in the critically ill, although an SVM based application has already been described by the authors in an ICU setting for prediction of the tacrolimus blood concentration in post liver transplantation patients [5]. SVM acknowledged fewer variables as significant to make its prediction of hospital mortality: it used only 4 variables for both models. In most ICU databases, there is a high percentage of missing values, making modeling of these data more difficult. If a prediction model uses less variables, the chance of having a high percentage of missing values will be lower with, possibly, a more accurate prediction as a result. For example, in this study, the SVM model 2 only needed 4 input variables namely ventilation < 24 h, bone marrow transplantation, bacterial infection and pulse. From these four variables, three of them are readily available. From this, it can be argued to consider variables for model development which are usually available in most patients, thereby reducing the number of missing values. The use of fewer variables in a SVM prediction model in comparison with a MLR model, was also demonstrated in prior SVM research by the authors [5]. Table 5 demonstrates that the PPV of the MLR and SVM algorithm have similar values, although the NPV for the SVM algorithm is lower than in the MLR model. Worthwhile mentioning is the fact that both the CSSI-score developed by Groeger and co-workers [14,20] and the more general APACHE II score and SAPS II score, predicted hospital mortality less accurate than the studied MLR and SVM models and this in both the training and validation data sets. This could be expected due to the fact that the tested models in this study were validated on patient data of the same ICU. Indeed, a truly independent validation of the findings of this study should be performed on separate data sets in different ICU's. A frequent problem with risk prediction models, especially prognostic models that have not been recently developed, is the weakening calibration of the model [21]. This problem can be dealt with by implementing a locally developed risk prediction model that can be updated over time, ideally in an automated way, e.g. by retraining the SVM algorithm or other artificial intelligence learning algorithms such as artificial neural networks by excluding one month of data at the start of the time series analysis and adding the last month's data [22]. The conclusion of equivalence in discrimination performance between the MLR and SVM results did not change when validating these methods with a 10-fold cross validation, in addition to the train and validation cohort methodology that was reported in the manuscript. The authors only reported the results obtained by a train and validation cohort methodology due to the overall acceptance of this methodology in medical community. In practice, SVM technology could be incorporated – after thorough validation – as an intelligent agent into the intensive care information systems and hence give decision support to the ICU clinician. This implementation of SVM technology in the ICU will be the subject of future research by this study group. While no discriminative model is capable of predicting the outcome of any individual patient and although some studies show the equivalence of the prognostic capacities of ICU clinicians [21] in comparison with the accuracies of risk prediction models, these locally developed models can, when well validated, give the ICU clinicians a perspective from which the care for the individual critically ill patient can only benefit.

## Conclusion

The discriminative power of both the MLR and SVM models was good. No statistically significant differences were found in discriminative power between MLR and SVM for prediction of hospital mortality in critically ill patients with haematological malignancies.

## Key messages

• Logistic regression is still the current standard in ICU prognostic modeling.

• New artificial intelligence methods are emerging for classification or prediction purposes in the ICU.

• The Support Vector Machine (SVM) algorithm has been proven to be a good classifier and prediction method in diverse scientific research areas.

• The accuracy for predicting hospital mortality by SVM is comparable to that of logistic regression prediction.

• SVM has the possibility – after further validation – to improve patient care in the near future by facilitating data modeling in the ICU.

## Abbreviations

ACC: accuracy; APACHE score: Acute Physiology and Chronic Health Evaluation score; AUC: Area Under the Receiver Operating Characteristic curve; CSSI: score Cancer Specific Severity of Illness score; ICU: Intensive Care Unit; LR: logistic regression; MLR: Multiple Logistic Regression; NPV: negative predictive value; PPV: positive predictive value; ROC: Receiver Operating Characteristic curve; SAPS: Simplified Acute Physiology Score; SN: sensitivity; SP: specificity; SVM: Support Vector Machine.

## Competing interests

The authors declare that they have no competing interests.

## Authors' contributions

JD, DB and FDT were responsible for the study design and they assume overall responsibility. The data acquisition was performed by DB. Data preprocessing was performed by SVL. TV and DB performed the statistical analysis. All authors were responsible for the interpretation of data. TV drafted the manuscript, all authors contributed to the final manuscript.

## Pre-publication history

The pre-publication history for this paper can be accessed here:


